# Insulin-like peptide has antagonistic pleiotropic effects on male combat traits and survival traits in an armed beetle

**DOI:** 10.1242/jeb.251318

**Published:** 2026-01-19

**Authors:** Takumi Kato, Chiho Yoshimine, Haruna Fujioka, Masako Katsuki, Kensuke Okada, Yasukazu Okada

**Affiliations:** ^1^ Graduate School of Science, Nagoya University, Nagoya, 464-8602, Japan; ^2^ Graduate School of Science, Tokyo Metropolitan University, Minamiosawa, 192-0397, Japan; ^3^ Graduate School of Environmental, Natural Science and Technology, Okayama University, Okayama, 700-8530, Japan

**Keywords:** Phenotypic plasticity, Sexual selection, Trade-off

## Abstract

The expression of sexually selected traits, such as exaggerated weapons and ornaments, often entails trade-offs against life-history traits. While phenotypic trade-offs are well documented, the underlying molecular physiological mechanisms remain largely unexplored. In this study, we investigated the potential role of an insulin-like peptide, ILP2, in mediating the trade-off between sexually selected combat traits and survival traits in the broad-horned flour beetle, *Gnatocerus cornutus*. RNA interference (RNAi)-mediated knockdown (KD) of *ILP2* during larval stages resulted in a reduction in the development of mandibular horns and overall body size. Interestingly, *ILP2* KD males had increased lipid storage and enhanced starvation tolerance, indicating a shift in resource allocation from sexually selected traits to survival traits. Behaviorally, *ILP2* KD males showed decreased locomotor activity and reduced aggression, leading to lower combat success. These findings suggest that ILP2 functions as a key mediator in the allocation of resources between combat and survival traits, highlighting its pleiotropic effects on morphology, metabolism and behavior. Our study provides novel insights into the molecular physiological mechanisms underlying life-history trade-offs associated with sexually selected traits.

## Teaser

**Summary:** The insulin-like peptide ILP2 mediates the resource allocation trade-off between combat traits and survival in *Gnatocerus cornutus*, shifting investment from weapons to starvation resistance.

## INTRODUCTION

Sexually selected exaggerated weapons and ornaments increase fitness of males by raising the chance of access to females (Andersson, 1994; Emlen, 2008, 2014; Shuster and Wade, 2019). Because these traits are generally costly to develop, their growth is highly sensitive to the nutritional state of the bearers (Emlen et al., 2012; but see Somjee, 2021; Somjee et al., 2021). Insulin/insulin-like signaling (IIS) is known to be a major mechanism that regulates the conditional expression of exaggerated characters (Emlen et al., 2012; Lavine et al., 2015). In deer, insulin-like growth factor 1 acts as the growth factor of antlers (Gu et al., 2007; Suttie et al., 1985). In a weaponed beetle, insulin-like peptide 2 (ILP2) regulates the conditional growth of mandibular horns (Okada et al., 2019). Although different factors of IIS are involved in weapon exaggeration in different species (InR: Emlen et al., 2012; Foxo: Casasa and Moczek, 2018; Rohner et al., 2023), IIS is one of the major regulatory pathways of weapon exaggeration.

Beside sexual traits, IIS often plays a multimodal, pleiotropic role in the regulation of growth, metabolism, reproduction and lifespan (Blüher et al., 2003; Clancy et al., 2001; Holzenberger et al., 2003; McElwee et al., 2004). In mammals and fruit flies, IIS is known to control overall body size (Hyun, 2013), and IIS has been implicated in regulating aging and longevity in various taxa ranging from nematodes to mice (Blüher et al., 2003; Clancy et al., 2001; Holzenberger et al., 2003; McElwee et al., 2004). In fruits flies, IIS-mediated metabolic regulation is associated with fat storage and starvation tolerance (Slaidina et al., 2009). Considering the multifunctionality of IIS, a deeper look into the involvement of IIS in non-sexual, life-history traits will be informative about the whole organismal function of IIS.

In terms of growth and metabolism, exaggerated traits are highly energy demanding to develop, maintain and use because of their extraordinary size and the intensity of their associated behaviors (e.g. courtship, fighting). Importantly, as the available resources in organisms are limited, such expensive traits often face trade-offs against life-history traits (Giannetti et al., 2024; Hill, 2011; Katsuki et al., 2012; McCullough et al., 2012; Simmons and Emlen, 2006). At the phenotypic level, such a trade-off relationship is widely known. However, the underlying physiological and molecular mechanisms responsible for these tradeoffs are much less well understood. Insect examples to date frequently involve shared trait responses to juvenile hormone (stalk-eyed fly: Fry, 2006; weaponed beetle: Okada et al., 2012; damselfly: Contreras-Garduño et al., 2011). In avians, testosterone increases aggression, influences parenting behavior and contributes to immunosuppression (Peters, 2007; Rosvall, 2013). Given the above-mentioned multifunctionality of IIS, insulin-like peptide (ILP) is a good candidate mediator of trade-offs between secondary sexual traits and life-history traits.

**Fig. 1.**
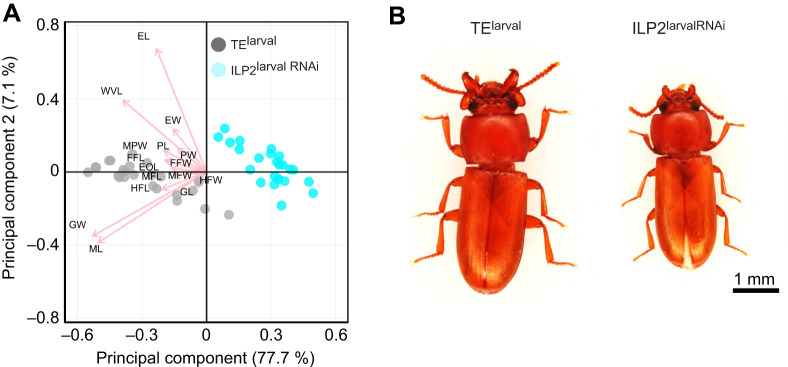
**Overall phenotypic changes caused by *ILP2* KD in *Gnatocerus cornutus*.** Principal component analysis (PCA) was performed based on the data of 16 morphological traits. Arrows indicate the factor loadings of each trait (see also Table S2). In the plot, gray represents the control group (TE^larval^) and light blue represents the *ILP2* KD group (ILP2^larvalRNAi^). ML, mandibular horn length; GW, gena width; EOL, eye outline length; EL, elytra length; EW, elytra width; PL, prothorax length; PW, prothorax width; MPW, maximum prothorax width; FFL, forefemur length; MFL, mid-femur length; HFL, hindfemur length; FFW, forefemur width; MFW, mid-femur width; HFW, hindfemur width; GL, genital length; WVL, wing vein length. (B) Representative phenotypes of control and *ILP2* KD males.

Males of the broad-horned flour beetle *Gnatocerus cornutus* have an enlarged ‘mandibular horn’ that is used as a weapon in male–male combat (Fig. 1; Okada et al., 2006; Vendl et al., 2018). In this species, one specific type of insulin-like peptide (GcorILP2) is the growth factor of this weapon (Okada et al., 2019). Given the multiple roles of IIS, we predicted that GcorILP2 might also have regulatory functions in non-weapon traits such as organ size, body size, metabolism and behavior. Specifically, we predicted that IIS may contribute to the trade-off between life-history traits and exaggerated traits. In this study, we investigated the wide physiological functions of ILP2, using RNA interference (RNAi)-mediated knockdown (KD) of *ILP2*.

## MATERIALS AND METHODS

### Broad-horned flour beetle (*Gnatocerus cornutus*)

The stock population of *Gnatocerus cornutus* Fabricius 1798 originated from wild-caught adults in Miyazaki prefecture, Japan, and has been maintained in the laboratory for over 50 years. The stock was reared in whole-wheat flour enriched with 5% brewer's yeast (EBIOS, Asahi Beer), in a plastic container (65 mm diameter, 105 mm height) under 14 h:10 h light:dark photoperiod at 25°C.

### RNAi-mediated *ILP2* knockdown

Double-stranded RNA (dsRNA) synthesis was conducted as described previously (Okada et al., 2019). Briefly, total RNA from larvae was reverse-transcribed into cDNA and used as the template for PCR. dsRNA was generated from the obtained amplicon using the AmpliScribe T7-Flash Transcription Kit (Lucigen). To consider potential off-target effects, we performed a protein alignment of all known *G. cornutus* ILP sequences using MAFFT version 7.490 (Katoh and Standley, 2013) in Geneious Prime software (version 2025.09) (Biomatter), and confirmed that the region of ILP2 targeted for RNAi has low similarity to any other ILPs (Fig. S1). Furthermore, a previous report has already shown that there are no conserved domains among the *G. cornutus* ILPs (Okada et al., 2019). Additionally, the primer sequences (Table S1) were searched against the transcript database of *G. cornutus* (Sugiyama et al., 2023) using BLASTN, to confirm target specificity.

For combat assays, in order to distinguish the effects of *ILP2* KD on morphology and behavior, we made two treatments (i.e. larval RNAi and adult RNAi). For *ILP2* KD targeting both adult morphology and physiology, final instar larvae were randomly selected from the stock culture and subjected to dsRNA injection with 46 ng of dsRNA diluted in TE buffer (23 nl). The injected larvae were kept in the isolated, unfed condition in 24-well plates. In this procedure, after larval isolation from the stock culture, prepupation occurs in 3–4 days and adult eclosion occurs in approximately 14 days (Ozawa et al., 2015). The effect of larval RNAi lasts until the adult stage (Fig. S2). As a control, TE buffer alone was injected (Sugiyama et al., 2023). These larval RNAi treatment beetles are referred to as ILP2^larvalRNAi^ and TE^larval^, respectively. For *ILP2* KD targeting only adult physiology, adults just after eclosion were injected with 46 ng of dsRNA diluted in TE buffer. Because adult morphology is fixed after eclosion in holometabolous insects such as this beetle, this treatment affects physiology and behavior but not external morphology. As a control, TE buffer alone was injected. These adult RNAi treatment beetles are referred to as ILP2^adultRNAi^ and TE^adult^, respectively. The knockdown efficiency was validated by qPCR using the KAPA SYBR^®^ Fast qPCR Kit (Kapa Biosystems) in individuals 2 weeks after adult eclosion (ILP2^larvalRNAi^ *n*=9, TE^larval^ *n*=9, ILP2^adultRNAi^ *n*=8, TE^adult^ *n*=5; Fig. S1).

We injected the solution into the joint between the dorsal abdominal segments of larvae and adults using Nanoject3 (Drummond Scientific) under CO_2_ anesthetization. The primers used for dsRNA synthesis and qPCR are listed in Table S1.

### Morphological measurements

Sixteen body parts (mandibular horn length, gena width, eye outline length, prothorax width, maximum prothorax width, prothorax length, elytra width, elytra length, wing vein length, genitalia length, forefemur length, forefemur width, mid-femur length, mid-femur width, hindfemur length, hindfemur width; see Fig. S2 for details) were measured from images photographed by a digital microscope (Leica microsystems, Leica LED3000 SLI) using ImageJ to the nearest 1 µm (*n*=24 per treatment). As in previous studies (Okada et al., 2019; Ozawa et al., 2016), elytra width was used as an index of body size.

### Measurement of adult fat content

The beetles subjected to the larval RNAi treatment (ILP2^larvalRNAi^ and TE^larval^, respectively) were frozen and killed at −20°C immediately after adult eclosion and placed in a dry heat sterilizer at 65°C for at least 24 h. Total dry mass was determined by electronic balance (Mettler Toledo, MS105DU). After dry mass measurement, samples were soaked in 0.5 ml of 2:1 chloroform:methanol mixture in a 2 ml glass vial and shaken for at least 2 days to remove lipids (Barnes and Blackstock, 1973). After lipid removal, individuals were placed back into the dry heat sterilizer at 65°C for at least 24 h, and total dry mass was determined again. The difference between the dry mass before and after chloroform:methanol treatment was used as the fat content (*n*=24 per treatment).

### Locomotor activity

Male beetles were placed individually in a 3.5 cm Petri dish on which a 3.5 cm piece of drawing paper was placed so that they could freely walk in the arena. Video (1 frame s^−1^) was captured with a web camera (Logitech; HD Pro Webcam C920t) between 13:00 h and 17:00 h for 1 h. From the videos, we tracked the location of the target individuals using a previously developed hand-written tracking program (Fujioka et al., 2017) with macOS Catalina (version 10.15.7). The distance traveled in 1 h was calculated from the coordinates. Males sampled just after eclosion (0 days) and at 14 days old were subjected to this analysis (*n*=18 per treatment).

### Combat assay

We observed combat behavior between pairs of males placed on circles of drawing paper (1.5 cm diameter) set in the bottom of a glass vial (1.5 cm in diameter and 3 cm high) and the interaction was observed for 30 min (Fig. 4A). A flowchart of the fighting behavior is shown in Fig. 4B. We detected the male that attacked first as an index of combat motivation. When no attack was observed from either individual, it was considered as ‘no combat’. If combat occurred, the individual that continued to chase the other beetle was designated as the winner and the individual that continued to run away was designated as the loser. When an attack was observed but the winner was still not determined for 30 min, the fight was considered a draw. ILP2^larvalRNAi^, TE^larval^, ILP2^adultRNAi^ and TE^adult^ males were subjected to this analysis when they were 14 days old, as they need 2 weeks to attain sexual maturity (Katsuki et al., 2012; Okada et al., 2020). Each treated male was paired against a non-injected wild type (WT) male, and this process was replicated 36 times (i.e. 36 independent trials) for each treatment. In order to minimize the effects of body size on fight outcome, contestant males were weighed on an electronic balance, and pairs of males with a mass difference less than 10% were used. To enable individual identification, a small dot of enamel paint (Tamiya, Inc.) was applied to the elytra (Fig. 4A). For each contest, the two males were introduced into the combat arena simultaneously to avoid any owner/resident effects. Observations were made under light conditions in a laboratory maintained at 24–28°C.

### Starvation tolerance

Survival under a food-deprived condition was measured for males from the TE^larval^ and ILP2^larvalRNAi^ treatments, which had been reared individually from the final larval instar, in 24-well plates lined on the bottom with 1.5 cm diameter drawing paper. To avoid water deprivation, 10 µl of water was dripped every 3 days. The day of adult emergence was taken as day 0, and the number of days until death was visually observed daily at 13:00 h (*n*=32 per treatment).

### Statistical analysis

Statistical analyses were performed using R version 4.5.1 (http://www.R-project.org/). The appropriate statistical test was chosen after checking the data for normality with the Shapiro–Wilk test and visually confirming the normality of the residuals using *Q–Q* plots. In morphological analysis, elytra width was used as a body size index as in previous studies (Okada et al., 2019; Ozawa et al., 2016). The data were not log-transformed as they met the assumptions for the statistical models used.

## RESULTS

### Function of ILP2 in organ and whole-body growth

To clarify the effect of ILP2 on adult trait formation, morphometric analysis was performed on control (TE^larval^) and ILP2^larvalRNAi^ males. We examined the effect of *ILP2* KD on organ size. Principal component analysis (PCA), which was based on a covariance matrix**,** showed that mandibular horn length (ML) and gena width strongly contributed to the first principal component (PC1, 77.7%) (Fig. 1A; Table S2). The factor loadings of each trait on the PC1 axis were all negative, indicating that a small PC1 means a large overall body size with pronounced ML and gena width. ILP2^larvalRNAi^ males are located on the right side, consistent with *ILP2* KD causing whole-body miniaturization, particularly in the weapon and its supporting head structures (Fig. 1B).

### Fat content in body

**Fig. 2.**
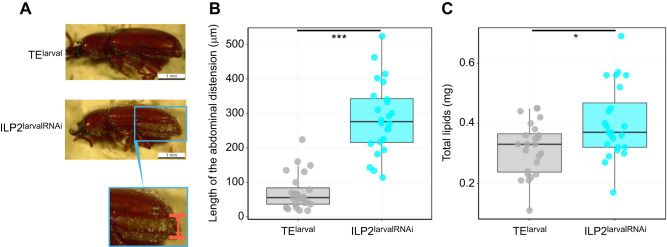
**Effects of *ILP2* KD on abdominal morphology and lipid content.** (A) The protrusion of the abdominal tergite beyond the elytral margin in *ILP2* KD *G. cornutus* (ILP2^larvalRNAi^) versus controls (TE^larval^), measured as the distance between the red lines (inset). (B) Changes in the exposed length of the abdominal tergite between *ILP2* KD and control individuals. Statistical analysis was performed using the Wilcoxon rank sum test (***P<0.001). (C) Comparison of total lipid content between control and *ILP2* KD individuals. Statistical analysis was performed using Student's *t*-test (*P<0.05). Box plots in B and C show median, upper and lower quartiles and 1.5× the interquartile range; circles are individual data points.

Despite their small body size, males injected with dsRNA as larvae (larval ILP2^larvalRNAi^) emerged as adults with ‘bulgy’ abdomens that were characterized by ventral and posterior distension, such that their abdomens often did not fit into the elytra (Fig. 2A). As there was no significant difference in the length of the elytra (ANCOVA, treatment, *F*_2,45_=30.14, *P*=0.7438; treatment×elytra width, *F*_3,44_=21.17, *P*=0.1562), we interpret this as abdominal distension rather than elytra reduction (Fig. S3, Table S3). Therefore, we measured the length of the protrusion of the abdominal dorsal margin from the elytra margin (Fig. 2A) and confirmed that the abdomen was enlarged (Wilcoxon rank sum test, *W*=560, *P*=2.16×10^−8^; Fig. 2B). In order to investigate the physiological characteristics of larval ILP2^larvalRNAi^ males, we measured the amount of fat in the whole body. The result shows that the amount of fat was increased in ILP2^larvalRNAi^ males (Student's *t*-test, *t*_46_*=*−2.8474*, P=*0.0066; Fig. 2C).

### Locomotor activity

**Fig. 3.**
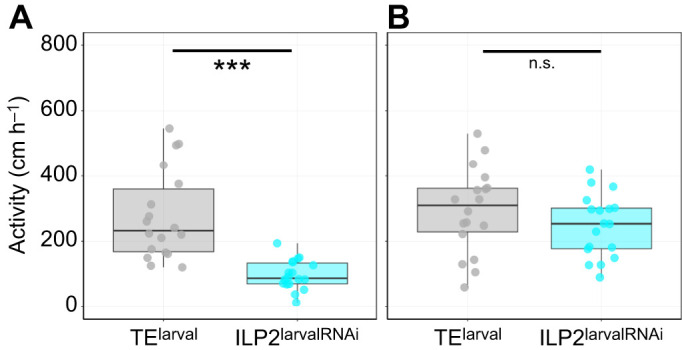
**Effects of *ILP2* KD on locomotor activity.** (A) Walking distance per hour of adults measured to assess the effect of ILP2^larvalRNAi^ on locomotor activity (0 days after eclosion). Statistical analysis was performed using Welch's *t*-test (***P<0.001). (B) Walking distance per hour of adults measured to assess the effect of ILP2^larvalRNAi^ on locomotor activity (14 days after eclosion). Statistical analysis was performed using Student's *t*-test (n.s., not significant).

To investigate the effect of ILP2 on locomotor activity, we measured the walking activity of adult males following *ILP2* KD. ILP2^larvalRNAi^ males at 0 days post-eclosion showed significantly reduced walking distance per hour (Fig. 3A; Wilcoxon rank sum test, *W*=17, *P*=2.67×10^−7^). There were no significant differences in walking activity in 14-day-old males (Fig. 3B; Student's *t*-test, *t*_34_=1.2219, *P*=0.23).

### Combat behavior

**Fig. 4.**
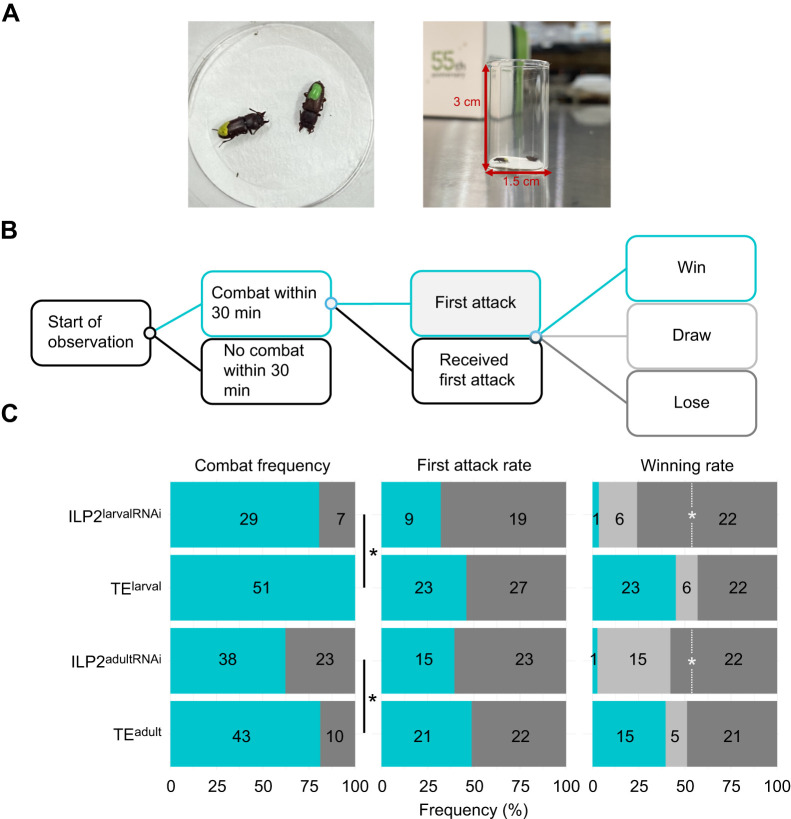
**Effects of *ILP2* knockdown (KD) on male fighting behavior.** (A) Left: *G. cornutus* males marked with enamel paint for individual identification. Right: the arena used for the combat assay. (B) Schematic diagram of the behavioral sequence used to evaluate combat performance. (C) Comparison of fighting behavior among the treatment groups. Results are shown using the same color coding as in the behavioral sequence (B). The difference of combat frequency was analyzed using Fisher's exact probability test. The frequency of first attacks and the winning rate were evaluated using binomial tests under the assumption that the expected value was 0.5 for size-matched combat (<10% mass difference). *P<0.05.

The effects on combat behavior were evaluated according to the behavioral sequence depicted in Fig. 4B. ILP2^larvalRNAi^ males engaged in combat at a lower frequency compared with TE^larval^ males (Fig. 4C; Fisher's exact probability test *P*=0.0014). In this experiment, fights were staged between size-matched individuals (<10% mass difference); thus, the expected values for the frequency of first attack and the winning rate were each 0.5. The observed frequency of first attacks did not differ from 0.5 for either ILP2^larvalRNAi^ males or TE^larval^ males (Fig. 4C; binomial test, ILP2^larvalRNAi^, *P*=0.087; TE^larval^, *P*=0.67). The winning rate of control males (TE^larval^) also did not differ from 0.5 (Fig. 4C; *P*=1, binomial test). However the winning rate of ILP2^larvalRNAi^ males was significantly less than 0.5 (Fig. 4C; *P*<0.001).

To evaluate the pure behavioral effect of *ILP2* KD, we also used ILP2^adultRNAi^ males and TE^adult^ males in combat experiments. The results were similar to those obtained with the larval RNAi treatment group, both in frequency of combat and in winning rate (Fig. 4C; Fisher's exact probability test, *P*=0.0014; binomial test, *P*<0.001, respectively), suggesting that *ILP2* KD at the adult stage is sufficient to cause the decline in combat behavior.

### Starvation tolerance

**Fig. 5.**
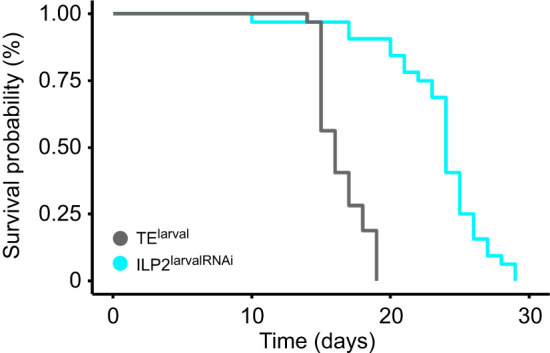
***ILP2* KD increases starvation tolerance.** Kaplan–Meier survival curves under food-deprived, water-only conditions.

As one aspect of life-history traits, we examined starvation tolerance under a food-deprived, water-only condition. The results revealed that ILP2^larvalRNAi^ significantly increased starvation tolerance (Fig. 5; log-rank test, χ^2^_3_=56.2, *P*=7.0×10^−14^). ILP2^larvalRNAi^ males survived about 23 days (23.5±0.67), which is 43.2% longer than TE^larval^ controls (16.4±0.29).

## DISCUSSION

Our study showed the antagonistic pleiotropic effect of ILP2 on combat traits (mandibular horn and combat behavior) and life-history traits (locomotor activity, lipid content and starvation tolerance). ILP KD decreased weapon growth and reduced aggressiveness and combat success, while at the same time increasing lipid storage and starvation tolerance. This suggests that ILP2 is not just a growth factor of weapons but also plays roles in the regulation of overall body size, behavior and physiology. Generally, sexually selected exaggerated weapons and ornaments and their associated behaviors (i.e. combat, display) are energy demanding and therefore their bearers often sacrifice allocation to other life-history traits (Emlen, 2001; O'Brien et al., 2019). As a result, the trade-offs between sexually selected traits and life-history traits are common outcomes of sexual trait evolution (Bonduriansky et al., 2008; Tidière et al., 2015). Despite the phenomenological understanding of such trade-offs, we currently know little about the mechanism of this trade-off. Our study showed that ILP2 is the mediator of one such trade-off between sexually selected combat traits and life-history traits.

*ILP2* KD caused a significant size reduction in the major sexual traits such as mandibular horn length and gena but also reduced overall body size (Fig. 1A). Interestingly, these *ILP2* KD males also metamorphosed into adults with greater amounts of lipids stored inside their bodies (Fig. 2C). Generally, in holometabolous insects, the lipid and protein stored in larval fat body are used as ‘building blocks’ for adult organ growth during metamorphosis (Arrese and Soulages, 2010; Burmester and Scheller, 1999). In *ILP2* KD males, the resources that would have been used for adult traits were invested less in sexual traits and more in fat storage during metamorphosis. Given that the insect fat body is mostly located in the abdomen, the ‘bulgy’ abdomen of KD males (Fig. 2A,B) probably reflects the increased amount of stored lipid.

In addition to morphological changes, *ILP2* KD also caused behavioral changes. First, locomotor activity was decreased in *ILP2* KD males, though this effect was limited to larval *ILP2* KD males and not observed in adult *ILP2* KD males (Fig. 3). This result may be attributed to a reduction in the size of the legs due to *ILP2* KD (Fig. S3, Table S3) or to the developmental delay or deficiency of uninvestigated traits, such as muscle and cuticle. Despite the limitation to the larval *ILP2* KD phenotype, ILP2 has a function in locomotor activity. Second, in *ILP2* KD males, combat performance was reduced. *ILP2* KD males initiated combat less often, suggesting that the motivation to fight was suppressed in these males. More importantly, *ILP2* KD resulted in a low winning rate for both ILP2^larvalRNAi^ and ILP2^adultRNAi^ males. In this study, we performed larval *ILP2* KD (ILP2^larvalRNAi^) and adult *ILP2* KD (ILP2^larvalRNAi^), therefore enabling us to distinguish morphological from behavioral effects. The decline in combat ability occurred in both ILP2^larvalRNAi^ and ILP2^adultRNAi^ males, indicating that ILP2 has a pure behavioral function, increasing aggression in adult males. In summary, ILP KD males are less active, more energy-saving individuals.

As one aspect of life-history traits, we investigated adult starvation tolerance. *ILP2* KD males lived 43.2% longer than control males under unfed conditions (Fig. 5), suggesting that ILP2 (and the weapon growth and fighting behavior it induces) normally shortens lifespan and reduces the starvation tolerance of males. Additionally, *ILP2* KD males stored more lipids and showed decreased locomotor activity. Several studies have suggested that maintaining sexually selected weapons may involve metabolic costs (Somjee, 2021; Somjee et al., 2018; Tullis and Straube, 2017), and our results provide a robust empirical example. *ILP2* KD males were behaviorally energy saving and physiologically energy rich. Thus, the increased starvation tolerance we observed in *ILP2* KD males should be attributed to abundant energy reserves and an energy-saving metabolic state.

These findings collectively propose that ILP2 acts as a key mediator of the trade-off between sexually selected traits and survival-related traits in *G. cornutus*. By promoting the development of exaggerated combat traits such as the mandibular horn and increasing combat activity, ILP2 enhances male competitiveness (Figs 2 and 4). However, these advantages may come at the cost of reduced energy storage and starvation resistance, indicating a modification in resource allocation pattern. This aligns with the broader concept that sexually selected traits often incur survival costs, indicating that ILP2 may help tune how resources are allocated between sexually selected and life-history traits.

It remains unclear whether the observed phenotypic effects are a ‘direct pleiotropic effect’ of ILP2 or a ‘hierarchical pleiotropic effect’ mediated by the reduction in body size. Therefore, further research is needed to disentangle the direct and indirect effects of ILP2.

In this study, we discovered that ILP2 has antagonistic pleiotropic effects that increase male investment in combat traits (weapon growth, aggressive behavior) while at the same time reducing lipid storage and starvation tolerance. From an evolutionary point of view, we propose the following scenario. In the ancestor before the acquisition of an enlarged male weapon, ILP2 may have acted as a general regulator of adult organ growth during metamorphosis and coordinated adult metabolism and behavioral activities. The organ growth and behavioral (aggression) function of ILP2 were probably co-opted during the evolution of the sexually selected male weapon, resulting in increased allocation to mandible growth in larval males and increased aggression and overall activity in adult males. Here, we show that this sex-specific change in weapon growth also resulted in significant metabolic and survival costs to the males, and our results suggest this is an antagonistic pleiotropic consequence of these diverse life-history traits all being regulated by the same physiological pathway (IIS).

## Supplementary Material

10.1242/jexbio.251318_sup1 Supplementary information
