# Supplementary material for: Insulin-like peptide has antagonistic pleiotropic effects on male combat traits and survival traits in an armed beetle
Source: J Exp Biol. 2026 Jan 19;229(2):jeb251318. doi: 10.1242/jeb.251318 (PMC12863297; doi:10.1242/jeb.251318)
Supplement: Supplementary information [file jexbio-229-251318-s1.pdf]

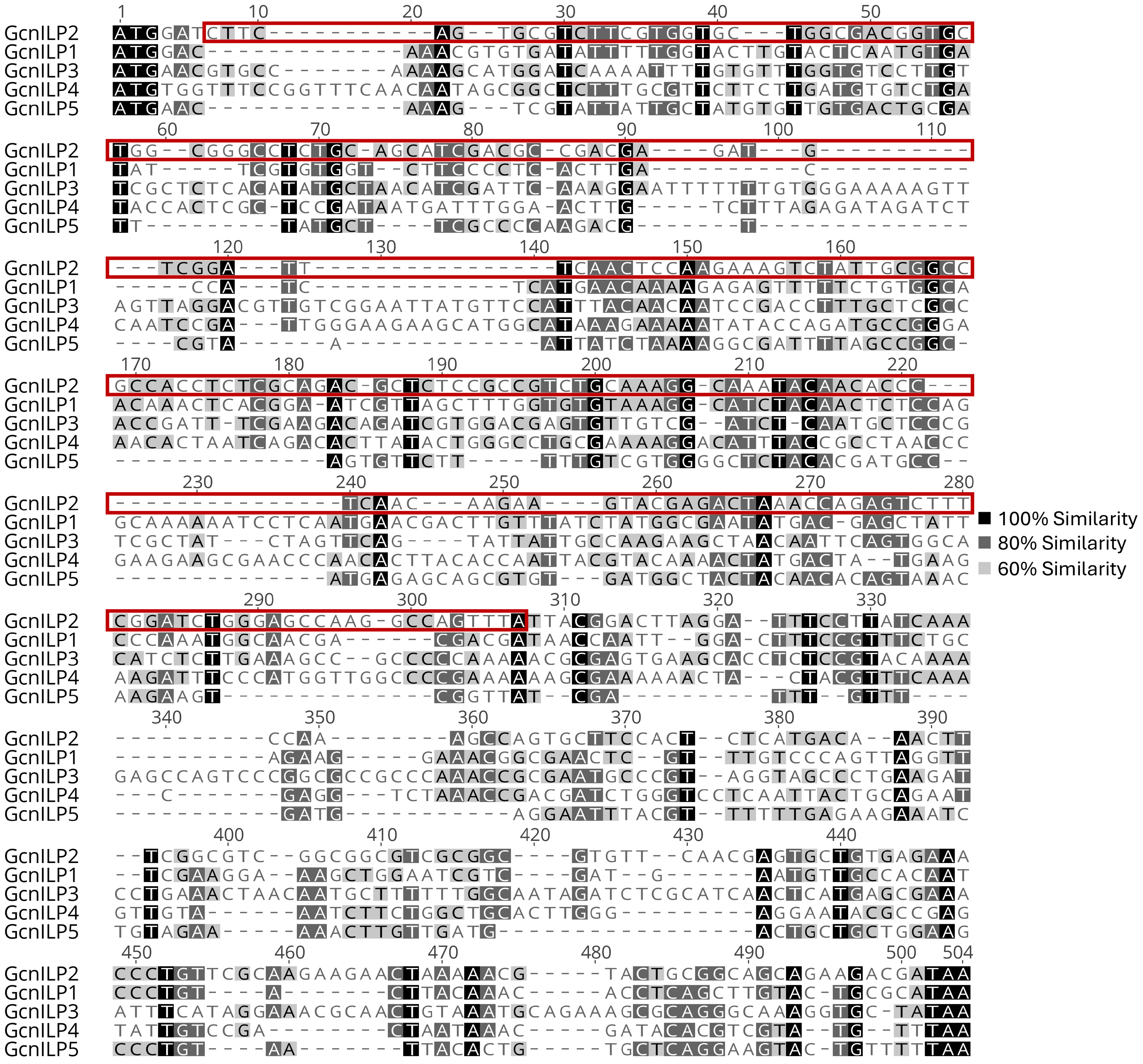

Fig. S1. Gene alignment of *G.cornutus* ILPs

Alignment of the ILPs. Regions with amino acid similarity are highlighted. The region of ILP2 targeted for knockdown in this study is indicated by a red box.

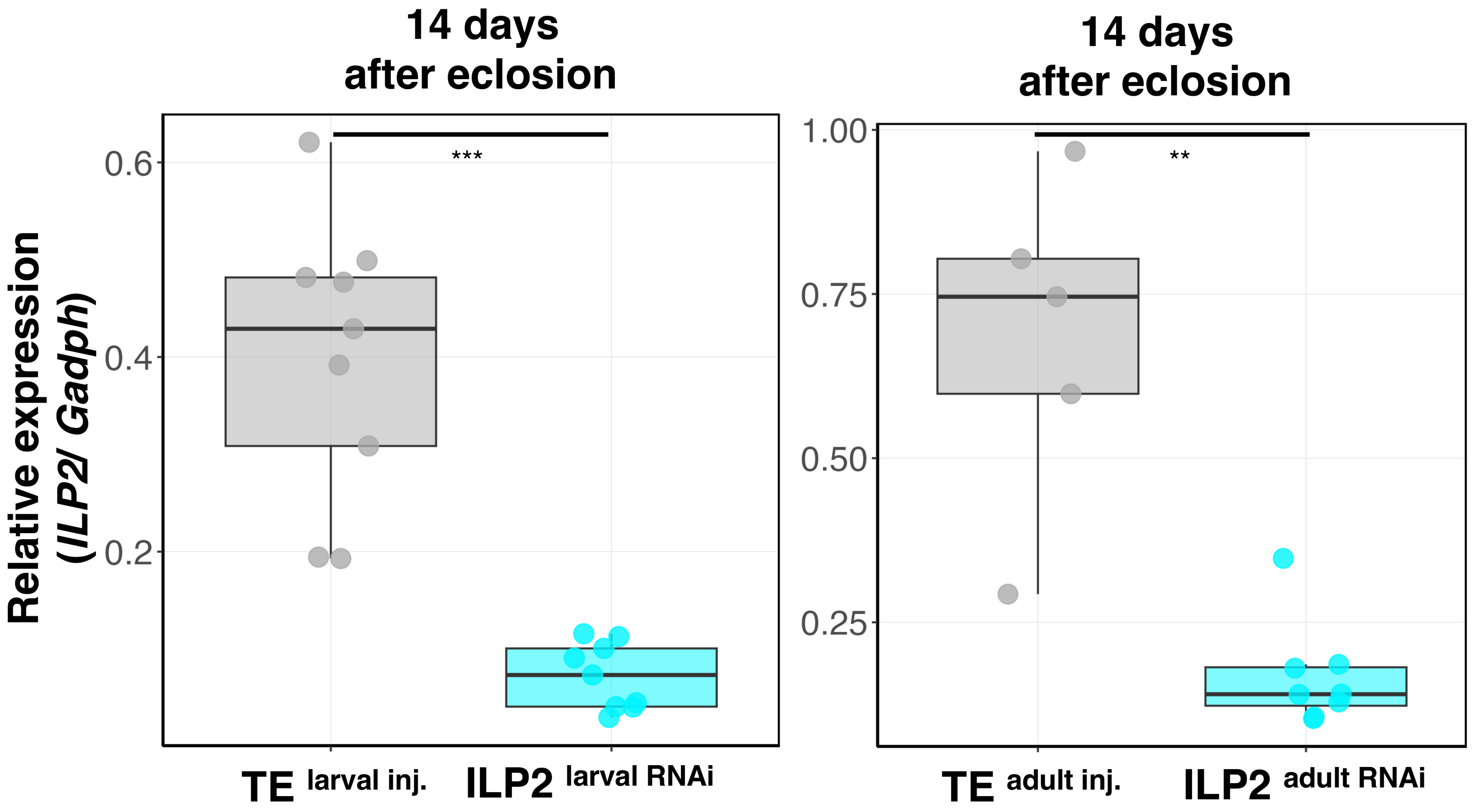

Fig. S2. Efficiency of RNAi.
ILP2 expression levels were measured using individuals two weeks after adult eclosion. For the larval RNAi condition, Welch's *t*-test was performed ($t = 6.6458$, $df = 8.8915$, ***$p < 0.001$). For the adult RNAi condition, the Wilcoxon rank sum test was used ($W = 39$, **$p < 0.01$). These results confirm the effectiveness of RNAi in both treatment conditions.

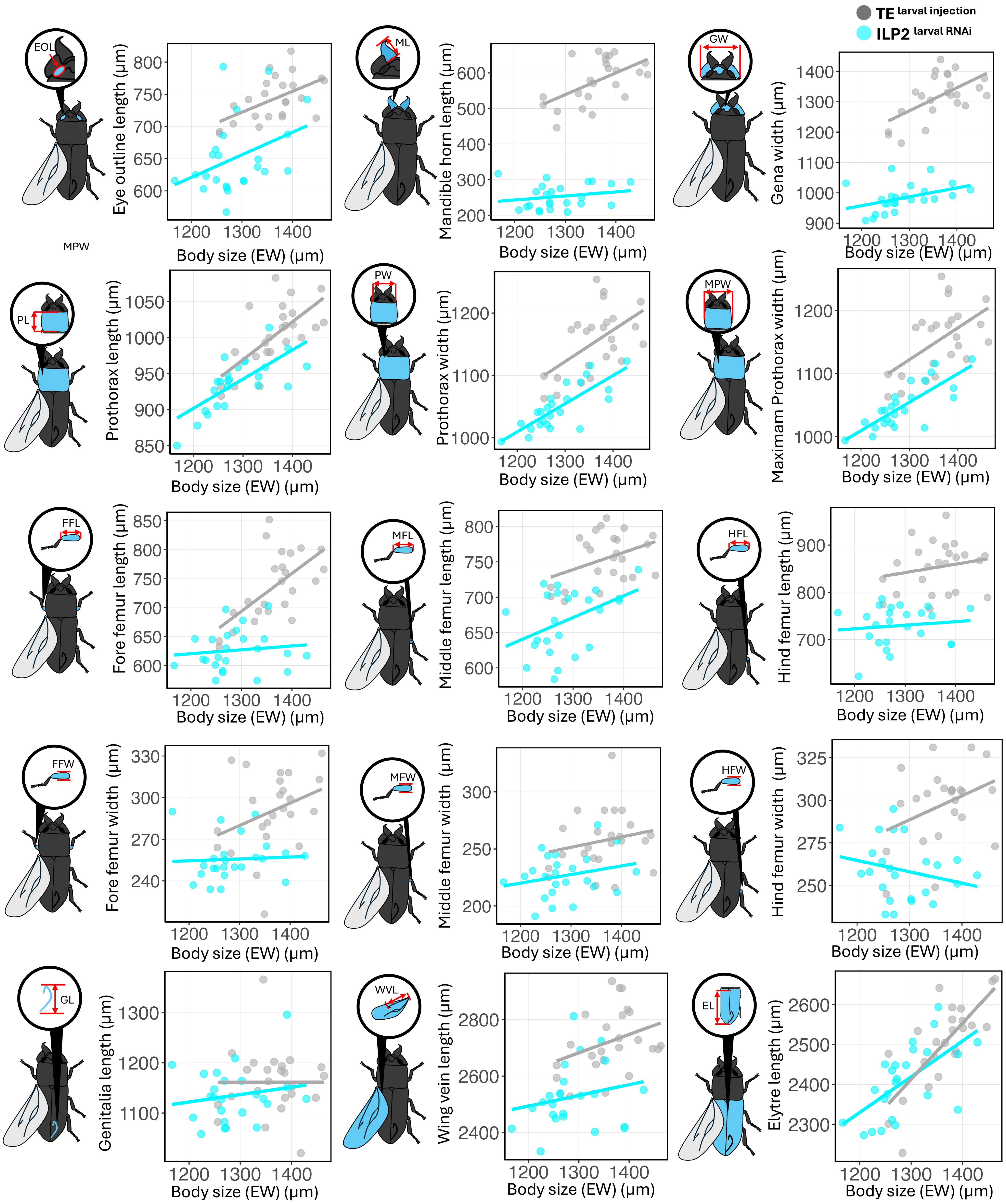

Fig. S3. Overall phenotypic changes caused by *ILP2* KD.

Effects of *ILP2* KD on adult males morphology. Landmarks used for each morphological measurement are shown in the illustration. Statistical analysis was conducted using ANCOVA; detailed results are presented in Table S3.

TableS1. Primer sequences for dsRNA synthesis and qPCR

**Gene**	**RNAi primer ID**	**RNAi primer sequence**
**ILP2**	T7GnaILPa6-221LEFT	TCTTCAGTGCGTCTTCGTGG
	T7GnaILPa6-221RIGHT	ATAAACTGGCCTTGGCTCCC
**Gene**	**qPCR primer ID**	**qPCR primer sequence**
**ILP2**	ILPa-qPCR-271-366F	ATGACAAACTTTCGGCGTCG
	ILPa-qPCR-271-366R	GCTGCCGCAGTACGTTTTTA
**gapdh**	gapdh-F	ATTTCCAACGCTTCTTGCAC
	gapdh-R	CCATCACGCCATAATTTTCC

Journal of Experimental Biology • Supplementary information

Table S2. Factor loadings in PCA analysis.

Traits	PC1	PC2
ML	-0.5037802	-0.3867548
GW	-0.5303976	-0.3503665
EOL	-0.1612596	0.02008406
PW	-0.1941722	0.06513541
MPW	-0.1988686	0.11895894
PL	-0.1344232	0.13204555
EW	-0.1542294	0.23427387
EL	-0.2327672	0.67316019
WVL	-0.3884937	0.39261604
FFL	-0.1909875	0.06369476
FFW	-0.0626333	-0.0039095
MFL	-0.1500754	-0.0212369
MFW	-0.0536995	-0.0259482
HFL	-0.2060786	-0.0946733
HFW	-0.0572075	-0.0933195
GL	-0.0558294	-0.0799148

Table S3. Effects of ILP2 KD on trait sizes.

Trait	Sample size		Effect(treatment)		Effect(Treatment * elytra width)	
	TE	ILP2KD	F (2,45)	P	F (3,44)	P
mandible length (ML)	24	24	287	P <0.001	209.8	P <0.05
gena width (GW)	24	24	243.7	P <0.001	171.9	0.07498
eye outline length (EOL)	24	24	30.72	P <0.001	20.03	0.9088
prothroax width (PW)	24	24	68.48	P <0.001	44.76	0.7641
maximam prothroax width (MPW)	24	24	51.51	P <0.001	33.83	0.6346
prothroax length (PL)	24	24	49.6	P <0.001	33.06	0.4143
elytra length (EL)	24	24	30.14	0.7438	21.27	0.1562
wing vein length (WVL)	24	24	21.12	P <0.001	13.93	0.6191
hind femur width (HFW)	24	24	21.91	P <0.001	17.37	P <0.05
